# Unconscious Reading

**Published:** 1882-05

**Authors:** 


### UNCONSCIOUS READING.

F. W. Harper sends to the *Spectator* the following letter:

A story which I have often heard from my father about his old college contemporary and intimate friend, Fearon Fallows, afterward Astronomer-Royal at the Cape of Good Hope—"Herschel, Peacock, Fallows," will recur readily to ears familiar with the rhythm of old Cambridge triposes—shows strikingly how knowledge far more startling than that mentioned by Mr. Griffiths may be received by the waking mind, it should seem, unconsciously, and yet may afterward work itself during sleep into most vivid consciousness.

One morning, Fallows—then a Johnian undergraduate, and working as Johnian candidates for the highest honors did, and doubtless do, work—was found in a state of the utmost excitement. He had "seen an apparition." An old friend and neighbor of his down in Cumberland had appeared to him in the night dripping wet and had told him that on such a day he had been drowned.

Undergraduate hearers received the story with incredulous laughter. In due course, however, letters from Cumberland came confirming it, and the laughers were silenced and confounded.

But some weeks afterward, a friend waiting in Fallows' rooms till their owner should be ready for a "constitutional," took up a newspaper which lay half-hidden under a heap of mathematical papers, and exclaimed, "Why Fallows, here's a full account in this newspaper of your friend's drowning." "Eh, what?" said Fallows, "*I* have seen no such newspaper." On examination it was found that the newspaper must have reached Mr. Fallows before the night of the apparition, and there was no doubt at all that, absorbed in working his mathematics, he had opened it unconsciously, and had read in it the startling intelligence of his friend's death unconsciously also.

Unconscious reading, adds the *Spectator*, which with many persons is a confirmed habit, is the explanation of a good many tricks of memory, as well as of some dreams, and perhaps, a great many plagiarisms.

